# KappaBle fluorescent reporter mice enable low-background single-cell detection of NF-κB transcriptional activity in vivo

**DOI:** 10.1038/s41385-022-00525-8

**Published:** 2022-05-19

**Authors:** Luigi Tortola, Federica Piattini, Annika Hausmann, Franziska Ampenberger, Esther Rosenwald, Sebastian Heer, Wolf-Dietrich Hardt, Thomas Rülicke, Jan Kisielow, Manfred Kopf

**Affiliations:** 1 Department of Biology, Institute of Molecular Health Sciences, ETH, Zurich, Switzerland; 2 Department of Biology, Institute of Microbiology, ETH, Zurich, Switzerland; 3 Department of Biomedical Sciences, University of Veterinary Medicine, Vienna, Austria

## Abstract

Nuclear factor-κB (NF-κB) is a transcription factor with a key role in a great variety of cellular processes from embryonic development to immunity, the outcome of which depends on the fine-tuning of NF-κB activity. The development of sensitive and faithful reporter systems to accurately monitor the activation status of this transcription factor is therefore desirable. To address this need, over the years a number of different approaches have been used to generate NF-κB reporter mice, which can be broadly subdivided into bioluminescence- and fluorescence-based systems. While the former enables whole-body visualization of the activation status of NF-κB, the latter have the potential to allow the analysis of NF-κB activity at single-cell level. However, fluorescence-based reporters frequently show poor sensitivity and excessive background or are incompatible with high-throughput flow cytometric analysis. In this work we describe the generation and analysis of ROSA26 knock-in NF-κB reporter (KappaBle) mice containing a destabilized EGFP, which showed sensitive, dynamic, and faithful monitoring of NF-κB transcriptional activity at the single-cell level of various cell types during inflammatory and infectious diseases.

## Introduction

Nuclear factor-κB (NF-κB) encompasses a family of evolutionarily conserved transcription factors^1,2^. Originally discovered as a transcriptional regulator for immunoglobulin κ-light chain^3,4^, NF-κB is now known to control a variety of cellular processes including proliferation, differentiation, apoptosis, and tissue homeostasis, in many cell types. In addition, NF-κB is of great importance in immunity and inflammation. It controls the transcriptional responses downstream of cytokine receptors and pattern recognition receptors as well as B- and T-cell receptors^5^, and its deregulated activation is associated with the development of inflammatory pathologies.

The activity of NF-κB is regulated by members of a family of inhibitory subunits (IκB) that bind to it and prevent its nuclear translocation. Induction of NF-κB depends on the activation of upstream kinases of the IKK family that phosphorylate IκB, targeting it for ubiquitylation and proteasomal degradation. Upon release, NF-κB is free to migrate to the nucleus and act as a transcriptional activator. Two major branches of NF-κB signaling, namely the “canonical/classical” and “alternative” pathway can be distinguished. The canonical pathway relies on p65/p50 NF-κB heterodimers and is activated downstream of TLRs, as well as several cytokine receptors, including receptors of IL-1 family members and TNF. Whole-body abrogation of the canonical pathway results in embryonic death caused by TNF-mediated liver wasting indicating its essential role in embryonic development^6,7^. While the canonical pathway is dispensable for lymphocyte development, it prevents TNF-driven apoptosis during inflammatory responses^8–10^. Inversely, deregulated activation of the canonical pathway is associated with the development of immune pathologies^11,12^. The alternative pathway instead depends on RelB/p105 heterodimers and mediates signaling downstream of CD40 or LTβR, making it crucial for B cell responses and for the development of lymphoid structures^13–17^.

Because of the prominent role of NF-κB in a broad variety of cellular processes, several approaches have been undertaken to monitor its activity by generation of reporter constructs. The majority of available reporters are based on three different approaches including (i) ectopic expression of a fluorescently-labeled NF-κB subunit and microscopy-based analysis of its cellular localization^18^, (ii) ectopic expression of a fluorescently-labeled IκB and detection of fluorescence loss during activation-driven IκB degradation^19^; (iii) expression of a reporter protein (EGFP or luciferase) under the control of a NF-κB-dependent promoter^20–23^. The first two systems have proven efficient in some settings, but their major drawback is the reliance on the (over)expression of fluorescently tagged components of NF-κB signaling, which may affect the functionality of the signaling protein and potentially alter cell physiology. These approaches also limit the analysis to a single member of the NF-κB (or IκB) family at a time, so that the dynamics of activation of other family members are inevitably overlooked. With respect to the first strategy, the necessity of determining the subcellular localization of fluorescent NF-κB subunits additionally precludes flow cytometric analysis. Systems relying on the detection of NF-κB transcriptional activity via the expression of reporter genes such as fluorescent proteins or luciferase show a delay in detection compared to reporter strategies measuring earlier events in the NF-κB activation pathway, but also have the benefit of not interfering with upstream signaling. The use of fluorescent reporters such as EGFP would furthermore enable unbiased, high-throughput, single-cell analysis of heterogeneous cell mixtures by flow cytometry. However, the inherent stability and long half-life of these reporter proteins impede a faithful analysis of the fine dynamics of NF-κB activation. Accordingly, existing EGFP expression-based reporter mice suffer from high fluorescence background and low sensitivity, which limits the usability of these systems^22^. In this work, we tackle the inherent problems identified in previously generated reporter mice and describe the development of low background, sensitive, fluorescence-based reporter system for the dynamic detection of NF-κB transcriptional activity in vivo and the generation of ROSA26 knock-in “KappaBle” NF-κB reporter mice.

## Results

### Generation of a low-background fluorescent reporter for NF-κB using a self-inactivating retroviral vector

**Fig. 1:**
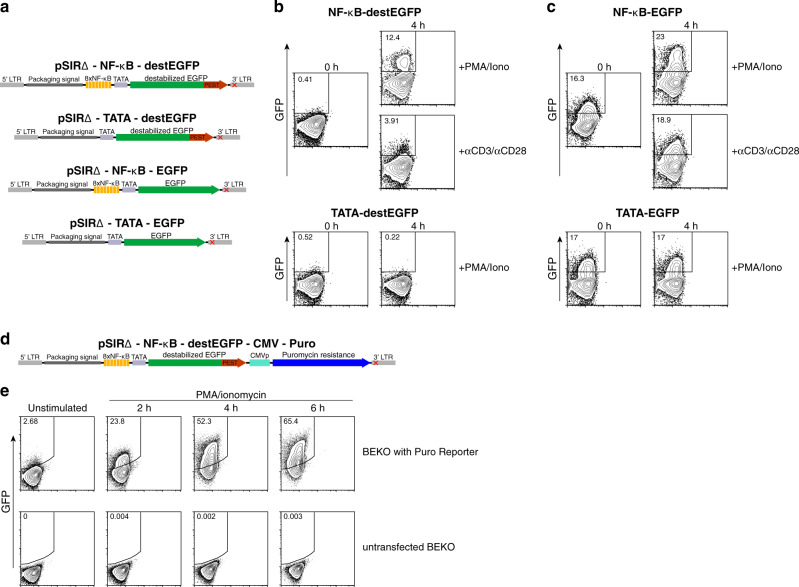
Sensitive, low-background detection of NF-κB activation with a retroviral reporter based on destabilized EGFP. **a** Schematic representation of pSIRΔ-NF-κB-destEGFP retroviral reporter vector and of the additional control constructs lacking NF-κB-binding sites and/or expressing conventional EGFP. LTR = long terminal repeat. The red cross in the 3’LTR indicates the mutation responsible for the inactivation of 5’LTR-dependent transcription upon integration into the host genome. **b** BEKO cells were infected with retroviral particles encoding NF-κB-destEGFP or TATA-destEGFP constructs. Cells were then stimulated with anti-CD3/anti-CD28 or with PMA and ionomycin and analyzed at the indicated time-points by flow cytometry to monitor GFP expression. **c** Flow cytometric analysis of BEKO cells carrying either the NF-κB-EGFP or the TATA-EGFP constructs after stimulation with PMA and ionomycin. **d** Schematic representation of pSIRΔ-NF-κB-destEGFP-CMV-Puro retroviral reporter vector. **e** BEKO cells were infected with retroviral particles encoding NF-κB-destEGFP-CMV-Puro. Following puromycin selection, cells were stimulated with PMA and ionomycin and analyzed at the indicated time-points by flow cytometry.

Aiming to generate a sensitive and low-background fluorescent reporter system for real-time analysis of NF-κB transcriptional activity, we based our strategy on two main aspects (Fig. 1a): first, to increase the sensitivity of NF-κB reporter system^24^, we used 8 NF-κB binding sites in the promoter region instead of 2–4 used in other reporter mice^22,23^; second, we used a destabilized version of EGFP containing a C-terminal PEST domain (destEGFP), which considerably reduces the half-life of EGFP and, hence, the background fluorescence in the absence of an activating stimulus. This should allow to better visualize the real-time dynamics of NF-κB activation^25^.

We incorporated the NF-κB-destEGFP construct into the backbone of a self-inactivating retrovirus (pSIRΔ). This vector enables delivery of the encoded reporter into dividing mouse cells and harbors a mutation that precludes 5’ long terminal repeat (LTR)-dependent expression of the reporter gene after integrating into the host genome^26–28^. To highlight the advantage of destEGFP as a reporter protein, we additionally generated a retroviral NF-κB reporter using conventional EGFP (NF-κB-EGFP). Lastly, for both reporter versions, we generated additional control constructs lacking the NF-κB binding cassettes, while maintaining the TATA box. These controls (named “TATA-destEGFP” and “TATA-EGFP”) serve to distinguish the fluorescent background originating from steady-state or cumulative NF-κB activation over time from that dependent on TATA box-dependent basal transcription. All reporter constructs were then introduced and tested in the BEKO thymoma cell line^29^. As shown in Fig. 1b, the NF-κB-destEGFP reporter led to virtually no fluorescence background in the absence of stimulation, while CD3/CD28 triggering or addition of PMA and ionomycin promptly induced destEGFP expression in BEKO cells. Notably, a substantial and comparable proportion of EGFP^+^ cells was observed in BEKO cells containing the NF-κB-EGFP and TATA-EGFP in the absence of stimulation indicating a high fluorescence background driven by the TATA box, which could be increased by PMA/Ionomycin, but not CD3/CD28 stimulation, in an NF-κB-dependent manner.

This data indicates that usage of a destabilized EGFP is mandatory to faithfully monitor the dynamics of NF-κB activation, with a pronounced difference between stimulated and non-stimulated cells.

In order to allow the use of this reporter construct for applications that require the selection of transduced cells, we also generated an additional construct encoding puromycin-N-acetyl-transferase that confers resistance to puromycin (Fig. 1d). Stimulation of BEKO cells transduced with pSIRΔ-NFkB-destEGFP-CMV-Puro and subsequently selected with puromycin led to robust upregulation of GFP by the majority of cells (Fig. 1e). These data show that our retroviral destEGFP-based reporter shows minimal background fluorescence, allowing for a much more faithful detection of NF-κB activation compared to reporters based on conventional EGFP.

### Analysis of NF-κB transcriptional activity in immune cells harboring the NF-κB-destEGFP reporter

**Fig. 2:**
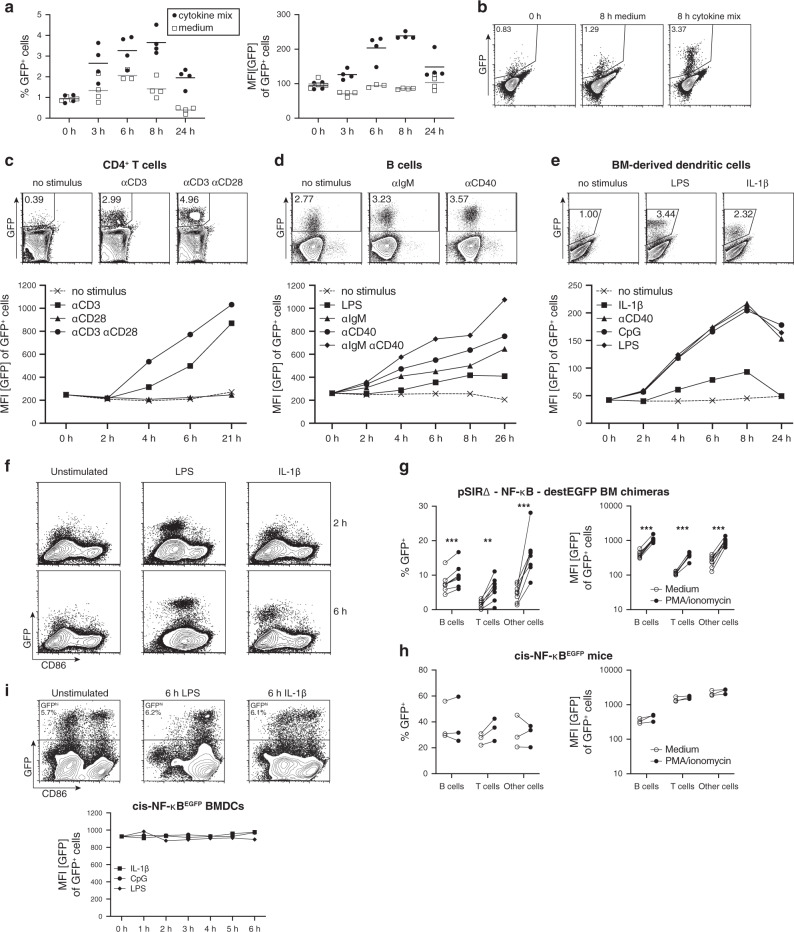
Faithful induction of destEGFP expression following activation of the canonical and alternative NF-κB pathways in primary immune cells. **a-b** BM cells infected with the retroviral destEGFP-based NF-κB reporter were treated with a cytokine mix consisting of IL-3, IL-6 and SCF or with fresh medium without cytokines and analyzed by flow cytometry at different time points. **a** Plots depict the percentage of GFP^+^ cells (left panel) and the mean fluorescence intensity (MFI) of GFP among GFP^+^ cells (right panel) at different timepoints after treatment. **b** Representative dot plots of the data shown in **a**. **c–e** Lethally irradiated mice were reconstituted with BM cells infected with pSIRΔ-NF-κB-destEGFP retrovirus to generate NF-κB reporter chimeras. Following reconstitution, we isolated CD4^+^ T cells (**c**), B cells (**d**) and differentiated BMDCs (**e**), and treated them with a variety of stimuli to trigger NF-κB activation. Upper panels show representative contour plots from flow cytometric analysis and the lower panels show the MFI of GFP among GFP^+^ cells. **f** BMDCs derived from the BM of NF-κB reporter chimeras were left untreated or stimulated with LPS or IL-1β. Contour plots show the expression of destEGFP and CD86 at the indicated time points. **g-h** Blood cells isolated from retroviral NF-κB reporter chimeras (**g**) or from cis-NF-κB^EGFP^ mice (**h**) were left untreated or stimulated with PMA and ionomycin to assess the upregulation of GFP following NF-κB activation. Each circle indicates an individual mouse, paired observations from the same animal are connected with a line. Statistical analysis was performed using paired *t* tests with Holm-Sidak correction for multiple comparisons. **i** BMDCs from cis-NF-κB^EGFP^ mice were left untreated or treated with LPS or IL-1β. Contour plots on the top show the expression of destEGFP and CD86 at the indicated time points, whereas the plot at the bottom shows MFI of GFP among GFP^+^ cells.

Experiments performed in the mouse thymoma-derived BEKO cell line indicated that our destEGFP-based retroviral reporter could provide efficient and dynamic detection of NF-κB activation following stimuli such as CD3 crosslinking or mitogen-mediated activation. To determine the efficiency of this reporter in primary mouse cells, we infected mouse bone marrow (BM) cells with pSIRΔ-NF-κB-destEGFP. Interestingly, when culturing the bone marrow cells after retroviral infection, we observed that the addition of medium containing a fresh cytokine cocktail including IL-3, IL-6, and SCF led to an increase in the number of fluorescent cells and in the level of destEGFP expression as compared to cells cultured with cytokine-free medium (Fig. 2a, b). Importantly, once the cytokines were consumed by the BM cells, the residual fluorescence rapidly decreased, confirming the crucial advantage of using destabilized EGFP as a reporter over conventional EGFP for the observation of the dynamics of NF-κB activation.

Bone marrow cells transduced with pSIRΔ-NF-κB-destEGFP were then transplanted into lethally irradiated mice to generate NF-κB reporter chimeras. We then purified CD4^+^ T cells and B cells from the spleens of reporter chimeras by MACS and stimulated them in vitro to study the dynamics of destEGFP expression. Similar to BEKO cells, primary CD4^+^ T cells showed no expression of destEGFP (and hence no activation of NF-κB) in steady state. However, expression was rapidly induced upon CD3 cross-linking. Treatment with anti-CD28 antibodies alone did not lead to activation of NF-κB, but it enhanced destEGFP upregulation when combined with anti-CD3 antibody (Fig. 2c). B cells already expressed destEGFP before the addition of any exogenous stimulus, indicating that even at steady state B cells have a certain level of NF-κB activation, in agreement with previously published data^4,30,31^. Importantly, stimulation of B cells with LPS, or by crosslinking IgM and/or CD40 on their surface, led to prompt upregulation of destEGFP expression as compared to non-stimulated cells (Fig. 2d).

To assess the functionality of the NF-κB reporter in myeloid cells, we differentiated BM-derived dendritic cells (BMDCs) from the bone marrow of reconstituted chimeric mice. We then stimulated BMDCs with LPS, CpG, anti-CD40 and IL-1β. DC activation led to destEGFP upregulation within 2 h with TLR stimulation or CD40 crosslinking, or 4 h when stimulating the cells with IL-1β (Fig. 2e). During the time-course, we also tracked the expression of the DC activation marker CD86 (Fig. 2f). Notably, destEGFP upregulation preceded the induction of CD86 following LPS treatment. As expected, in the case of IL-1β-driven NF-κB activation, destEGFP expression was induced in the absence of CD86 upregulation (Fig. 2f).

We next compared the sensitivity of our pSIRΔ-NF-κB-destEGFP reporter to a NF-κB fluorescent reporter mouse strain (i.e., cis-NF-κB^EGFP^) established previously^22^. The reporter cassettes of the two systems differ in the number of NF-κB binding sites (8 vs 3) and in the reporter protein (destabilized vs conventional EGFP). Stimulation of blood cells isolated from pSIRΔ-NF-κB-destEGFP retro-transgenic mice with PMA and ionomycin led to a clear upregulation of GFP (Fig. 2g) on B cells, T cells, and myeloid cells. Blood cells from cis-NF-κB^EGFP^ animals showed high background fluorescence in the absence of any stimulus, and in vitro stimulation with PMA and ionomycin did not result in further upregulation of GFP (Fig. 2h). Similarly, stimulation of cis-NF-κB^EGFP^ BMDCs with LPS or IL-1β did not upregulate GFP above levels of unstimulated cells (Fig. 2i).

Collectively, these data demonstrate that the retroviral destEGFP-based reporter can be employed to visualize the transcriptional activity of NF-κB in primary murine immune cells ex vivo by flow cytometry. The elevated number of NF-κB response elements in the promoter region and the use of destabilized EGFP as a reporter protein leads to a significant increase in sensitivity compared to previously generated fluorescent reporter animals. Furthermore, we also show that our reporter can successfully portray the activation of both major pathways of NF-κB signaling, i.e., the classical (for TLRs, IL-1R, BCR, and TCR) and the alternative pathway (CD40).

### Generation and characterization of KappaBle NF-κB reporter mice

**Fig. 3:**
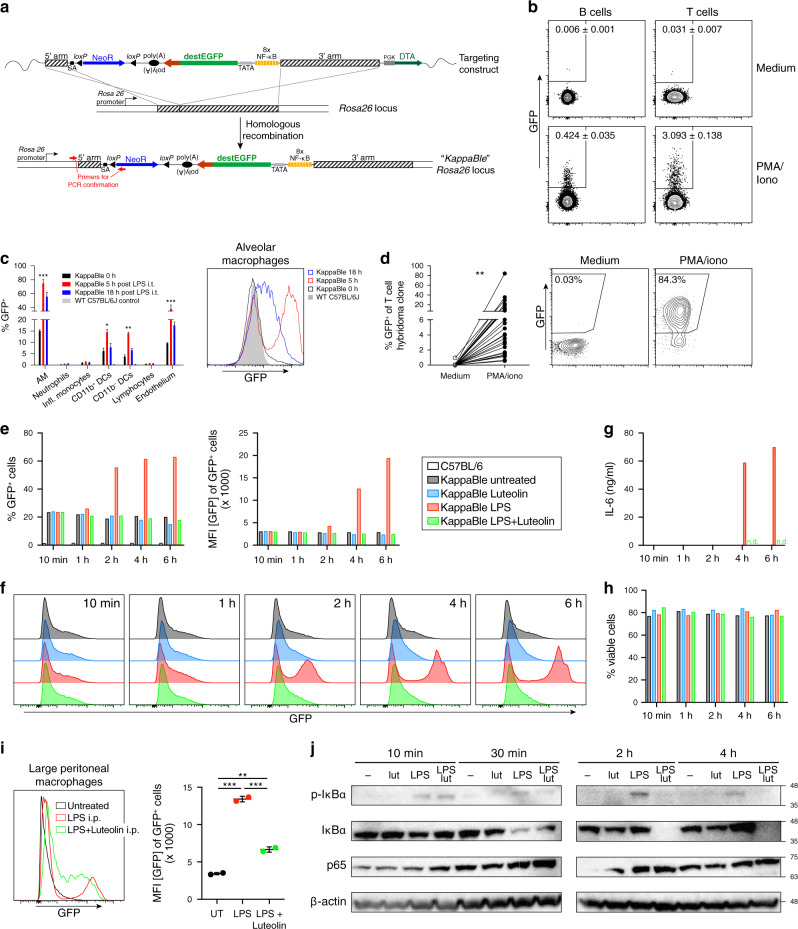
Generation and characterization of KappaBle NF-κB reporter mice. **a** Schematic representation of the vector generated to deliver the NF-κB reporter construct to the ROSA26 locus in ES cells and of the targeted “KappaBle” locus after homologous recombination. **b** Cytometry plots show destEGFP expression in B and T cells from the blood of KappaBle mice (*n* = 9) before and after in vitro stimulation with PMA and ionomycin. **c** KappaBle (C57BL/6J) and C57BL/6J control mice were treated with LPS intratracheally and cells from the lungs were analyzed by flow cytometry at the indicated time points after injection. Bar graph on the left shows % of GFP^+^ cells in the indicated populations and timepoints and histogram overlay on the right shows GFP expression in AMs at different timepoints. Statistical analysis was performed using two-way ANOVA with Dunnett correction for multiple comparisons (3 mice per group/timepoint). **d** Hybridomas were derived from KappaBle T cells. Individual hybridoma clones (*n* = 26) were left untreated or stimulated with PMA and ionomycin. Cells were then analyzed by flow cytometry. GFP expression is plotted on the left panel, paired observations from each individual clone are connected by a line (statistical analysis was performed with paired *t* test). The contour plot on the right shows expression of GFP^+^ by the most responsive KappaBle T cell hybridoma clone. **e**–**h** In vitro cultured AMs derived from KappaBle mice were left untreated or stimulated with LPS, in the presence or absence of NF-κB inhibitor luteolin. Cells were analyzed at the indicated timepoints. **e** Bargraphs depict % GFP^+^ cells (left) and MFI of GFP among GFP^+^ cells (right). **f** Histogram overlays show GFP expression in KappaBle AMs treated as indicated at the different timepoints. **g** IL-6 secretion from stimulated AMs at the different timepoints was measured by ELISA. **h** Percentage of viable (Sytox Red-negative) AMs with the different treatments at the indicated timepoints. **i** KappaBle mice received LPS i.p. 30 min after PBS or luteolin i.p. injection. 5 h later, cells from the peritoneal cavity were isolated and analyzed by flow cytometry. Large peritoneal macrophages were gated as CD45^+^ Ly6G^–^ CD11b^high^ F4/80^high^. Each symbol represents an individual mouse, statistical analysis was performed using one-way ANOVA with Tukey’s correction for multiple comparisons. **j** Western blots depict expression of IκBα (phosphoform and total), p65 and β-actin in AMs treated as indicated at the different timepoints. β-actin levels are shown as control. Uncropped images are depicted in Fig. S1.

The results presented in the previous sections show that our construct allows sensitive, quantitative and rapid detection of NF-κB activation in hematopoietic cells. Retro-transgenesis is a suitable solution to incorporate our reporter in immune cells, but in order to be able to track NF-κB transcriptional activity across all cell lineages, we decided to generate knock-in mice encoding the reporter construct in the ROSA26 locus^32^. After transfection of C57BL/6-derived ES cells and geneticin selection, integration of our targeting construct by homologous recombination was assessed by PCR on the short arm (Fig. 3a). Successfully targeted ES cells were used to generate “KappaBle” NF-κB reporter mice. Lymphocytes isolated from the blood of KappaBle mice (Fig. 3b) lacked background fluorescence, and stimulation with PMA and ionomycin lead to a significant and faithful upregulation of GFP, although only in a relatively small fraction of T cells (~3%) and B cells (~0.4%). Hetero- or homozygous expression of the KappaBle cassette yielded comparable results (data not shown). On the other hand, intratracheal administration of LPS led to striking upregulation of GFP in alveolar macrophages (AMs, >70%), DCs (~15%) and CD31^+^ endothelial cells (~40%) in the lungs 5 h post injection (Fig. 3c). Importantly, this GFP signal significantly faded 18 h after the administration, highlighting again the advantage of destEGFP as a reporter protein to enable dynamic measurement of NF-κB transcriptional activity. Considering that a large fraction (>65%) of BEKO thymoma cells containing pSIRΔ-NFkB-destEGFP-CMV-Puro potently upregulate GFP expression upon stimulation, we were wondering why only a small fraction of T cells (and B cells) from KappaBle mice upregulate GFP upon PMA/Ionomycin stimulation. To address this question, we generated KappaBle T cell hybridomas by fusion of T cells from the spleen of KappaBle mice with BW5147 cells. Interestingly, the resulting clones showed enormous range of maximal GFP responses following PMA/ionomycin stimulation, ranging from ~0.5 to >80% (Fig. 3d), indicating that random epigenetic changes associated with the fusion determine the accessibility of the reporter cassette to the transcriptional machinery. Nonetheless, background fluorescent in the absence of a stimulus was consistently extremely low across all T cell hybridoma clones, further confirming the specificity and low background of our reporter system.

To probe the specificity of GFP upregulation in KappaBle cells following stimulation, we stimulated in vitro cultured alveolar macrophages with LPS in the presence or absence of luteolin, a known inhibitor of NF-κB^33–36^ (Fig. 3e–h). While GFP was not upregulated within the first hour of stimulation, AMs showed a massive increase 2 to 6 h upon exposure to LPS (Fig. 3e,f), which was completely abrogated in the presence of luteolin (Fig. 3e, f). Accordingly, secretion of IL-6, a known target of NF-κB activation, was detected starting after 4 h of LPS stimulation and was blocked in the presence of luteolin (Fig. 3g). Importantly, luteolin did not affect survival of AMs within the timeframe of measurement (Fig. 3h). These data demonstrate that KappaBle cells are faithful and specific reporters of NF-κB transcriptional activity. Consistent with these in vitro results, administration of luteolin in vivo diminished GFP upregulation in KappaBle large peritoneal macrophages following i.p. injection of LPS (Fig. 3i).

To compare the kinetics of detection of NF-κB activity in cells from KappaBle mice, we determined expression and phosphorylation of IκBα in stimulated AMs by Western blot (Fig. 3j, Fig. S1). Expectedly, IκBα phosphorylation was detected already after 10 min and IκBα degradation started 30 min after stimulation by LPS. Total IκBα protein levels were restored after 2 h and IκBα phosphorylation was maintained up to 4 h in response to LPS. As previously reported^33^, treatment with luteolin diminished but did not abolish IκBα phosphorylation and degradation after 30 min. Interestingly, absence of IκBα protein in LPS/luteolin treated cells after 2–4 h indicated that luteolin interferes with NF-κB-dependent replenishment of IκBα protein, which is consistent with the finding that p65 transactivation induces IκBα transcription in a NF-κB autoregulatory pathway^37,38^.

Together, these data demonstrate the functionality and specificity of the NF-κB reporter cassette in KappaBle mice.

### Monitoring of NF-κB transcriptional activity in a variety of cells in the lung and intestine during inflammation

**Fig. 4:**
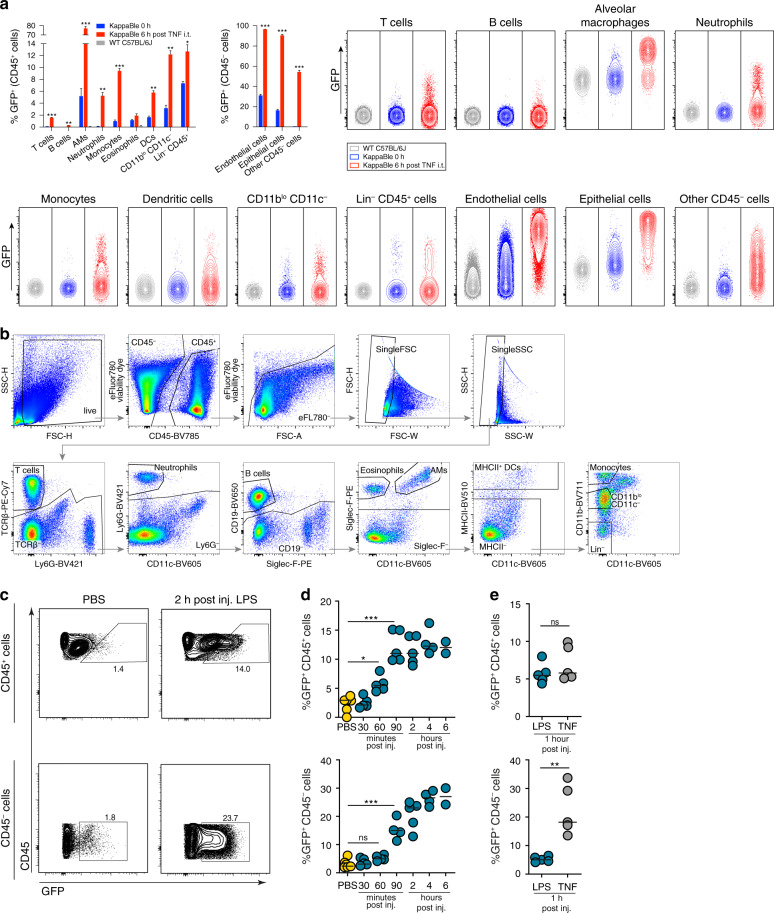
Detection of NF-κB transcriptional activity in KappaBle mice following in vivo administration of TNF and LPS. **a** KappaBle and WT C57BL/6J mice were treated with TNF intratracheally and analyzed by flow cytometry 6 h after injection. Bar graphs on the top left show the percentages of GFP^+^ cells among indicated populations of CD45^+^ and CD45^–^ cells, while contour plots on the right depict GFP expression in the indicated populations and groups. Statistical analysis was performed using paired *t* tests with Holm-Sidak correction for multiple comparisons (3 mice per group/timepoint). **b** Gating strategy for the flow cytometry data depicted in **a**. **c-d** KappaBle mice received LPS intravenously. At the indicated timepoints, animals were euthanized and cecal cells were isolated and analyzed by flow cytometry. **c** Representative dot plots depict GFP expression among CD45^+^ and CD45^–^ cecal cells 2 h after LPS or PBS administration. **d** Plots depict GFP expression among CD45^+^ (upper panel) and CD45^–^ cecal cells (lower panel) at different timepoints after LPS administration. **e** KappaBle mice received LPS or TNF intravenously. Cecal cells were isolated and analyzed by flow cytometry 1 h post injection. Plots depict GFP expression among CD45^+^ (upper panel) and CD45^–^ cecal cells (lower panel). Each symbol represents an individual mouse, statistical analysis was performed using one-way ANOVA with Tukey’s correction for multiple comparisons in **d** and unpaired *t* tests in **e**.

Receptors for TNF (TNFR1 and/or TNFR2) are broadly expressed by many cell types and are known to trigger NF-κB activation after engagement with their ligand. To visualize NF-κB transcriptional activity across different cell types in an inflammatory setting, we administered TNF by intratracheal instillation to the lungs of KappaBle mice and analyzed a variety of hematopoietic and non-hematopoietic cells isolated from the lungs 6 h later by flow cytometry (Fig. 4a, b). Similar to endotoxin exposure, we found a strong responsiveness of AM to TNF (>70% GFP^+^). Other immune cell types, including neutrophils, monocytes, DCs, CD11b^lo^ CD11c^–^ cells (predominantly NK cells) and Lin^–^ cells (including γδ T cells and ILCs) showed a sizable upregulation of GFP (5–15%) following exposure to TNF in vivo. B and T cells isolated from the lungs of KappaBle mice showed measurable and consistent upregulation of GFP after NF-κB activation, but only in a minority of cells, consistent with the observations in the blood. Eosinophils were ~1% GFP^+^ in untreated mice, and this percentage slightly increased following TNF administration.

Importantly, analysis of the non-immune CD45^–^ compartment in the lungs revealed that TNF triggered the upregulation of GFP in almost all EpCAM^+^ epithelial and CD31^+^ endothelial cells, as well as in ~50% of other CD45^–^ stromal cells.

We next studied the kinetics of GFP activation in the large intestines of KappaBle mice following intravenous injection of LPS or TNF. Consistent with a previous report using GFP-p65 NF-κB reporter mice^39^, injection of LPS led to the upregulation of GFP^+^ among CD45^+^ immune cells (maximal response achieved within 90 min) and then among CD45^–^ intestinal epithelial cells (maximal upregulation within 2 h, Fig. 4c, d), while the expression of GFP showed similar kinetics in both compartments after injection of TNF (Fig. 4e). Expectedly, NF-κB transcriptional activity in cecal CD45^–^ cells occurred with a delay and only in a fraction of cells in KappaBle mice compared to GFP-p65 reporter animals, in which nuclear translocation of GFP-p65 could be detected by two-photon microscopy in virtually all cecal epithelial cells within 1 h after LPS injection^39^, indicating that nuclear translocation of NF-κB is not necessarily reflecting transcriptional activation.

These data show that the NF-κB reporter in KappaBle animals displays minimal background and is functional across all immune cell types we analyzed, although the percentage of responding cells varies. Among CD45^–^ non-immune cells this percentage greatly increased and almost all epithelial and endothelial cells responded to NF-κB activation by upregulating GFP.

### Influenza-infected alveolar macrophages and lung epithelial cells show increased NF-κB activation

**Fig. 5:**
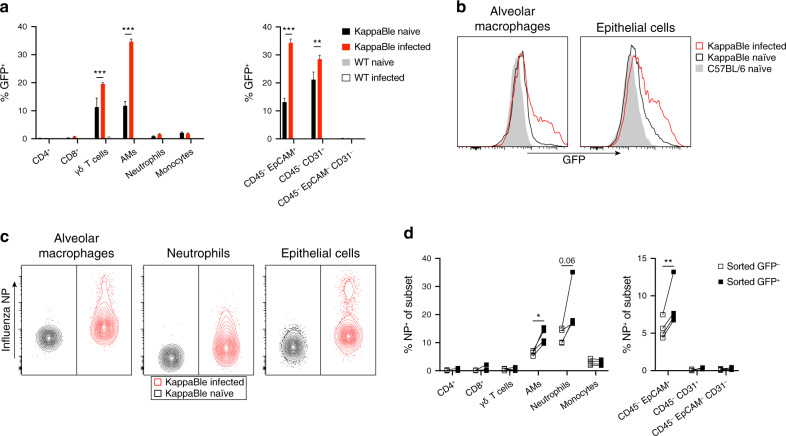
Analysis of NF-κB transcriptional activity in the lungs of influenza-infected KappaBle mice. KappaBle mice (*n* = 4) were infected with influenza A virus. On day 5 post infection lung cells were isolated and analyzed by flow cytometry. **a** Bar graphs show GFP expression in the indicated populations of CD45^+^ and CD45^–^ cells. Statistical analysis was performed using two-way ANOVA with Sidak correction for multiple comparisons. **b** Representative histogram overlays depict GFP expression in AMs and lung epithelial cells. **c** Lung cells were stained intracellularly for influenza NP to detect infected cells. Contour plots depict representative NP staining in AMs, neutrophils and lung epithelial cells. **d** NP staining was performed in sorted GFP^+^ and GFP^–^ cells from infected mice. Plots depict % NP^+^ among CD45^+^ (left panel) and CD45^–^ cell populations (right panel). Each symbol represents an individual mouse, paired observations from the same mouse are connected with a line. Statistical analysis was performed using paired *t* tests with Holm-Sidak correction for multiple comparisons.

We next studied the dynamics of NF-κB activation in the lungs of influenza-infected KappaBle mice. Analysis of immune cells 5 days post-infection showed a clear upregulation of GFP in AMs and γδ T cells compared to naïve mice (Fig. 5a, b). Among non-immune cells, both epithelial and endothelial cells showed significantly increased GFP expression following infection (Fig. 5a, b). We then wondered whether NF-κB activation correlated with whether a cell had been infected or not. Intracellular staining of influenza nucleoprotein (NP) showed that the virus predominantly infected (or was taken up by) AMs and neutrophils and a small proportion of epithelial cells (Fig. 5c). Interestingly, NP staining in sorted GFP^+^ and GFP^–^ cells showed that infected cells were significantly enriched among GFP^+^ AMs and GFP^+^ epithelial cells (Fig. 5d), although not every infected AM and epithelial cell was also GFP^+^, indicating that infection did not necessarily activate NF-κB. Neutrophils showed a similar, albeit statistically non-significant, trend. Moreover, although the frequency of GFP^+^ endothelial cells and γδ T cells increased during infection, NF-κB activation in these populations seems to be driven by inflammation rather than direct viral infection since they were negative for NP. These data highlight the potential of KappaBle mice for the unbiased analysis of NF-κB transcriptional activity during complex cellular responses in vivo.

## Discussion

NF-κB plays an essential role in a plethora of cellular processes. In order to efficiently track the activation of this transcription factor, we set out to generate a reporter construct based on GFP expression, to allow for rapid, high-throughput analysis by flow cytometry. Common problems encountered when using GFP as a reporter gene are the low sensitivity and the high levels of background fluorescence^40^. To overcome such obstacles, we generated a retroviral vector encoding a destabilized EGFP version with a short half-life under the control of multiple NF-κB binding sites. Expression of this vector in a thymoma cell line and primary mouse immune cells showed inducible and faithful expression of GFP when activating NF-κB with various stimuli, such as TLR ligands, CD3-crosslinking, or cytokines, and a low fluorescent background in the absence of a stimulus. Importantly, due to the overlapping reactivity of the response element with the various isoforms of NF-κB, we could efficiently detect activation of both the canonical pathway, induced for instance by TLR ligands or IL-1β, and of the alternative pathway, triggered by CD40 crosslinking, making our retroviral NF-κB reporter a powerful tool for the detection of NF-κB activation.

To allow the measurement of the dynamics of NF-κB activation also in non-hematopoietic cell lineages in vivo, we generated KappaBle NF-κB reporter mice. We decided against random transgenesis to avoid adverse integration effects and chose to introduce the reporter into the ROSA26 locus, which is accessible in most cell types^32^. Compared to the integration of a fluorescent reporter into the locus of a known endogenous NF-κB target gene, knock-in of a surrogate target gene allows to increase the faithfulness of the reporter by excluding the contribution of additional transcriptional regulators capable of binding to the promoter of an endogenous target gene, as well as its sensitivity by increasing the number of NF-κB response elements in the promoter region as reported^24^, improving the inefficient detection of NF-κB activity provided by mice harboring fewer response elements (such as cis-NF-κB^EGFP^ mice^22^). Analysis of hematopoietic cells from KappaBle mice showed that the reporter cassette was functional across all analyzed immune cell types and in vivo or in vitro stimulation with NF-κB-activating stimuli led to consistent upregulation of GFP. Treatment with NF-κB inhibitor luteolin effectively inhibited LPS-driven upregulation of endogenous NF-κB target genes including IL-6 and IkBα, as well as the expression of the designed surrogate target gene destEGFP in the reporter cassette, demonstrating the specificity of KappaBle reporter mice. However, the fraction of cells responding to NF-κB activation with the expression of GFP varied in the different cell types: after in vivo stimulation, lung resident AMs showed an almost universal response, while other myeloid cells (including DCs, neutrophils and monocytes) showed upregulation of GFP in a subset of cells (5–15%). Among lymphocytes, NK cells and γδ T cells/ILCs showed the most pronounced upregulation of GFP upon activation (>10%), while the fraction of GFP-expressing cells was relatively low among αβ T cells (~3%) and, in particular, B cells (<1%). This could be explained by epigenetic silencing of the reporter construct or expression of a repressor that binds to the NF-κB motif and prevents transcription in certain cell types. This hypothesis is supported by the analysis of hybridomas derived from KappaBle T cells, in which the maximal upregulation of GFP greatly varied among different clones.

Importantly, analysis of the non-immune compartment of KappaBle mice following in vivo NF-κB activation showed GFP upregulation by all lung epithelial and endothelial cells and by ~50% of remaining stromal cells, demonstrating the great potential of KappaBle mice for the study of NF-κB transcriptional activity in non-immune cells in vivo. To highlight the potential application of our reporter animals for the analysis of NF-κB in complex in vivo cellular processes, we investigated the activity of NF-κB in the lungs of influenza-infected KappaBle mice and found that it was primarily activated in virus-infected AMs and lung epithelial cells.

Currently available genetically engineered NF-κB reporter mouse models employ different NF-κB activity detection strategies. Each of these strategies has its own advantages and disadvantages and, most importantly, is suited for specific applications. Luciferase-based reporters allow in vivo bioluminescence imaging but are incompatible with single cell analysis. Reporters based on NF-κB-dependent expression of GFP are less sensitive than luciferase-based reporters but allow high-throughput multidimensional single cell analysis by flow cytometry. Reporter mice expressing a GFP-p65 fusion protein offer a great resolution of the early events of NF-κB activation (i.e., nuclear translocation of p65) by microscopy^39^, but preclude a high-throughput flow cytometric analysis and do not report activation of the alternative NF-κB pathway by focusing on p65 translocation. While selection of one type of reporter system over the other strictly depends on the scientific question and the specific methodology to employ, we show here that KappaBle mice allow faithful fluorescence-based detection of the transcriptional activity of NF-κB (both classical and alternative pathway), with a significant improvement in terms of background and sensitivity compared to previously existing reporter animals using an analogous strategy^22^.

In summary, we describe here the generation of a fluorescence-based, low-background reporter system for the study of NF-κB. Both the retroviral reporter and knock-in KappaBle reporter mice offer the possibility to track NF-κB transcriptional activity during complex processes in vivo or in vitro at the single cell level, making them useful tools for researchers studying the role of NF-κB in health and disease.

## Materials and methods

### Cloning of retroviral NF-κB reporter vector and of KappaBle targeting construct

The octameric NF-κB response element was generated by multimerizing a sequence encoding two distinct NF-κB binding sequences^24^, which was obtained by annealing of two synthetic oligonucleotides with complementary 5’ overhangs (sequences: 5ʹ-agc tTG GGG ACT TTC CGC GAG GGA TTT CAC CTA-3ʹ and 5ʹ-agc tTA GGT GAA ATC CCT CGC GGA AAG TCC CCA-3). The octamer was gel-purified, amplified by PCR with Platinum Taq polymerase (Invitrogen) using single stranded oligonucleotides as primers and finally re-purified from the gel. The product was cloned into p123TA vector and sequenced. The octamer was then extracted from the vector by BamHI (made blunt) and EcoRI digestion and cloned into pBluescript II-KS(+) digested with EcoRV and EcoRI to generate pBS NFκB. The IL-2 minimal promoter sequence containing a TATA-box was amplified from mouse genomic DNA with Pwo polymerase (synthesized and purified in-house as previously described^41^) using the primers 5ʹ-agt att ggt ctc gaa tt g CAT CGT GAC ACC CCC ATA TTA T-3ʹ and 5ʹ-agg gat ccA GGC AGC TCT TCA GCA TGG GAG-3ʹ^24^. The PCR product was digested with BsaI and EcoRI and cloned into pBS-NFκB cut with BamHI and EcoRI to yield pBS-NFκB-IL2mp. Destabilized EGFP (destEGFP) was extracted from the pd2EGFP vector and cloned into pBS-NFkB-IL2mp by using NotI and BamHI, generating pBS-NF-κB-destEGFP. The construct was sequenced and the NF-κB reporter cassette was transferred into pSIRΔ vector (Clontech) using XhoI and NotI. To generate the reporter construct carrying puromycin resistance (Fig. S1A, B), the cassette from pSIRΔ-NFkB-destEGFP was cloned into pSIRΔ-U6-CMV-Puro using XhoI and BglII.

To generate the KappaBle embryonic stem (ES) cell targeting construct, a synthetic double-stranded oligonucleotide containing a loxP site was first cloned into the pBS NF-κB reporter vector using SacII and NotI. The sequence of the oligonucleotide was 5ʹ-TAC CGC GGT CTC GAG GTC ATG CAT CAT AAC TTC GTA TAA TGT ATG CTA TAC GAA GTT ATT AAT AAA GAC GCG TCT CTA GAG CGG CCG CTA-3ʹ. Following this step, a 240-bp fragment from pHL-HH vector (courtesy of H. Fehling^42^) encoding additional polyadenylation signals was cloned using MluI and XbaI, yielding pBS NF-κB loxP polyA. The long arm of homology from pHL-HH was cut with Asp718 and AscI (blunt) and cloned into pBS NF-κB loxP polyA opened with Asp718 and SalI (blunt), generating pBS NF-κB loxP polyA LA. The reporter-LA cassette was then excised and cloned into pHL-HH by using NsiI and Asp718, to yield the final KappaBle targeting vector. All the enzymes used for restriction digestion were purchased from New England Biolabs or from Roche Applied Science.

### Mice

Mice with specific pathogen-free status according to FELASA were bred and maintained in individually ventilated cages at the ETH Phenomics Facility (Zurich, Switzerland). For experiments, age-matched mice in the age of 6–10 weeks were used. Swiss federal and local animal ethics committees approved the described animal experiments.

### Cell preparation

Single cell suspensions from the spleen were prepared by pressing the organ through 70 μm pore size strainers (BD Biosciences) in PBS 2% FCS. To obtain splenic dendritic cells, spleens were minced and digested 45 min with collagenase IV (300 units/mL) and DNaseI (200 units/mL, both enzymes from Worthington) before filtration through cell strainers. To isolate lung cells, IMDM medium supplemented with collagenase IV (600 units/mL) and DNaseI (200 units/mL) was introduced into the lungs of euthanized mice by cannulating the trachea. Perfused lungs were then digested for 45 min under agitation at 37 °C, followed by fine mincing and filtration through 70μm cell strainers. For the analysis of intratracheally-treated TNF-treated mice, lungs were digested using Liberase TM (Sigma, 50 µg/mL) and DNaseI (200units/mL) and processed using the gentleMACS dissociator (Miltenyi). Peritoneal cells were obtained by flushing the peritoneal cavity with 10 mL PBS 5 mM EDTA. Cells were washed once with PBS 2% FCS and subsequently stained. For some experiments, CD4^+^ T cells and B cells were MACS-sorted with anti-CD4 and anti-CD19 microbeads (Miltenyi), respectively. For the generation of bone marrow-derived dendritic cells (BMDCs), bone marrow (BM) cells were obtained from donor mice by flushing the femurs and the tibiae with PBS 2% FCS and cultured on bacterial, non-treated Petri dishes in RPMI medium supplemented with 10% FCS, HEPES, glutamine, penicillin/streptomycin and granulocyte-monocyte colony stimulating factor (GM-CSF, supernatant from X63-GMCSF cell line). Fresh medium was added on day 3, 6, and 8. For experiments, BMDCs were collected on day 9 by gentle pipetting from the culture plate.

### Retroviral transfection

Retroviral particles were assembled using the Phoenix packaging cell line^43^. Cells were seeded in complete IMDM (10% FCS, P/S, β-ME). About 5 min prior transfection, fresh medium containing 25 μM chloroquine was given to the cells. Separately, 50 μg of the retroviral construct were resuspended in 1752 μl of ddH_2_O, and 248 μl of a 2 M CaCl_2_ solution were added. After that, 2 ml of 2× HBS solution (50 mM HEPES, 10 mM KCl, 12 mM dextrose, 280 mM NaCl, 1.5 mM Na_2_HPO_4_, pH 7 ± 0.1) were added with a 5 ml pipette while vigorously aerating. This mix was then immediately added to the cells, which were then incubated for 8 h. After this time, fresh medium was added to the cells and the retroviral supernatant was collected 24 h later. This procedure was repeated once more before discarding the cells. Supernatant was stored at −80 °C till use. For retroviral transfection of bone marrow cells, donor mice were injected with 5-fluorouracil (150 μg/g bodyweight) intraperitoneally. Six days later, donor mice were sacrificed, the hind limbs removed and the bone marrow collected by flushing the femurs and the tibiae. Cells were cultured in a 24-well plate (500,000 cells/well) in complete IMDM supplemented with 6 ng/ml IL-6 (Sigma), 6 ng/ml IL-3 (eBioscience) and 10 ng/ml SCF (produced in-house). On the following day, retroviral supernatants were supplemented with 4 μg/ml polybrene and the cytokines from above, and distributed on the cultured BM cells. Spin infection was performed by centrifuging cells at 1800 rpm for 45 min, incubation for 1 h at 37 °C and repetition of the centrifugation after providing fresh retroviral soup. The whole procedure was then repeated the following day, before reconstitution of lethally irradiated recipient mice with at least 5 × 10^5^ transfected BM cells. For transfection of BEKO cells^29^, minor modifications of the protocol from above were used. No cytokines were supplemented in the medium and only one spin infection round (1800 rpm for 30 min) was performed.

### In vitro culture of alveolar macrophages

Fetal liver monocytes were collected and polarized to AM as described^44^. Briefly, fetal livers were isolated from E14.5-E17.5 pregnant females and digested with 2 mg/mL type IV collagenase (Worthington), 0.125 mg/mL DNaseI (Worthington) and 3% FCS in IMDM (Gibco) for 15 min at 37 °C and subsequently passed through a 70 µm strainer (Becton Dickinson). Red blood cells were lysed with homemade ACK lysis buffer for 5 min at RT. CD45^+^ cells were enriched using MACS sorting following manufacturer’s instructions. Fetal liver monocytes were cultivated in GlutaMAX supplemented RPMI (Gibco) containing 10% FCS, 10 mM Hepes, 50 µM b-Mercaptoethanol, 1% NaPyruvate, 100 U/mL Penicillin, 100 u/mL Streptomycin, 0.2 ng/mL hTGRb (Peprotech) and 30 ng/mL mGM-CSF (Peprotech) using untreated six-well plates. After 14 days, a homogeneous population of AM-like cells was obtained that can be cultured long-term without loss of AM identity.

### In vitro stimulations

For the experiments described in the manuscript, soluble stimuli were used at the following concentrations: lipopolysaccharide (LPS, InvivoGen) 500 ng/ml, CpG (InvivoGen) 100 nM, IL-1ß (ThermoFisher) 200 ng/ml, anti-CD40 (clone 1C10, Biolegend) 1 µg/ml, PMA (Sigma) 10^−7^M, ionomycin (Sigma) 1 μg/ml. For plate coating, anti-CD3ε (clone 145-2C11, Biolegend) and anti-CD28 (clone 37.51, Biolegend) were used at a concentration of 2 µg/ml, at and anti-IgM F(ab’)_2_ (Jackson Immunoresearch Laboratories) at 10 µg/ml. For the stimulation of in vitro cultured alveolar macrophages, cells were collected using 4 mM EDTA in PBS and counted. 1 × 10^5^ cells per well were plated in an untreated 96-well plate overnight and stimulated on the consecutive day with 400 ng/mL LPS and/or 100 µM luteolin (Sigma-Aldrich). At the end of the stimulation supernatant was carefully removed and frozen for ELISA analysis. Cells were detached using 4 mM EDTA in PBS, washed once and stained with 1:4000 SytoxRed in PBS 2% FCS before flow cytometric analysis.

### In vivo treatments and infections

For pulmonary treatments, 100 ng LPS suspended in 50 µL PBS were injected intratracheally and animals were analyzed 5 and 18 h post injection. TNF (Peprotech) was also administered intratracheally (1 µg in 50 µL PBS) and animals were analyzed 6 h later. For systemic administration, 5 µg ultrapure *Salmonella Typhimurium* LPS (kind gift of Otto Holst, Borstel, Germany) or 3 µg TNF (Peprotech) in 100 µl PBS were injected intravenously. For the stimulation of large peritoneal macrophages, 1 µg LPS was injected intraperitoneally (i.p.). 30 min after administration of luteolin (200 µg, i.p.). Influenza infection was performed by intratracheal administration of 500 pfu Influenza virus strain PR8 (A/Puerto Rico/34, H1N1). On day 5 post-infection, animals were euthanized and lung cells were isolated for analysis.

### ES cell culture and selection and blastocyst injection

LK1 C57BL/6J mouse ES cells^45^ were grown on a layer of irradiated neomycin-resistant EFs in ES medium (DMEM Glutamax supplemented with 1000 units/ml LIF, 15% ES grade FCS, 1 mM sodium pyruvate, MEM nonessential amino acids, penicillin/streptomycin, β-mercaptoethanol). On the day of transfection, ES cells were trypsinized and washed in PBS. 10^7^ cells ES cells were suspended in 800 µl transfection mix (HEPES 20 mM, pH7.0, NaCl 137 mM, KCl 5 mM, Na_2_HPO_4_ 0.7 mM, Glucose 6 mM, β-Mercaptoethanol 0.1 mM) containing 30 μg KappaBle targeting construct which had been linearized overnight with PvuI and purified by ethanol precipitation. This mix was then transferred to an electroporation cuvette (0.4 cm) and electroporated with 0.240 kV, 475 µF. Cells were incubated 10 min at room temperature, then transferred in ES medium and distributed on 3 culture dishes. Geneticin (175 μg/ml) was added for selection starting 1 day after transfection. Medium was changed daily until day 7, when colonies were picked and cultured in 96-well plates. Screening for homologous integration of the short arm was performed by a 2-round nested PCR on the ES DNA. The primers used for the first round were 5ʹ-CCT AAA GAA GAG GCT GTG CT-3ʹ (ExtRnd1), 5ʹ-CAT CAC AGC AGC CGA TTG TC-3ʹ (NeoRnd1), whereas those for the second round were 5ʹ-AGA GAG CCT CGG CTA GGT AG-3ʹ (ExtRnd2), 5ʹ- CAT AGC CGA ATA GCC TCT CC-3ʹ (NeoRnd2). The reaction was carried out using Platinum Taq polymerase, in the presence of 5% DMSO. Each round of PCR consisted of 30 cycles of 95 °C for 30 s, 56.5 for 30 s, 68 °C for 3 min. Of 36 picked clones, 6 were found to be positive and were further expanded and subsequently frozen in ES freezing medium (90% ES medium, 10% DMSO). 3 positive clones were karyotyped, microinjected into BALB/c blastocysts and transferred into the uterus horns of pseudopregnant surrogate mice. Chimeras were obtained and crossed to C57BL/6J mice. Twenty-one black offspring were screened for the presence of the neomycin resistance cassette, nine were found to be positive and named “KappaBle” mice.

### T cell hybridoma generation

Splenic T cells isolated from KappaBle mice were activated with plastic-bound anti-CD3ε and anti-CD28 Abs in the presence of mouse IL-2 for 2–3 days. Equal numbers of T cells and BW5147 cells were then fused using PEG-1500 and plated at limiting dilution in the presence of 100 mM hypoxanthine, 400 nM aminopterin, and 16 mM thymidine (HAT) and after selection single clones were isolated.

### Immunoblotting

1 × 10^6^ in vitro cultured AMs were plated in untreated 6-well plate overnight and stimulated on the next day. After stimulation cells were detached using 4 mM EDTA in PBS at 37 °C for 7 min, collected and washed once with PBS. Cells were then lysed on ice with RIPA buffer with protease and phosphatase inhibitor (Sigma-Aldrich) for 15 min. Protein concentrations were determined using the Pierce BCA Protein Assay Kit (Thermo Scientific). 30 µg proteins were denatured with Laemmli buffer at 95 °C for 5 min and fractioned by SDS-PAGE followed by wet-transfer to a polyvinylidene difluoride membrane using a transfer apparatus (Bio-Rad). Membranes were blocked with 4% nonfat milk in TBST (50 mM Tris, pH 8.0, 150 mM NaCL, 0.1% Tween20) for 45 min, washed and incubated with primary antibodies (anti-phospho-IκBα, Cell Signaling Technology, Cat# 9246; anti-IκBα, Cell Signaling Technology, Cat# 9242; anti-NF-κB p65, Biolegend, Cat# 622602) at 4 °C overnight. After washing, membranes were incubated for 1 h at RT with horseradish peroxidase-conjugated anti-rabbit (Southern Biotech, Cat# 4050-05) or anti-mouse (Southern Biotech, Cat# 1030-05) antibodies. For β-actin staining membranes were incubated with horseradish peroxidase-conjugated primary antibody (Sigma-Aldrich, Cat# A3854) and stained for 30 min at RT. Blots were washed and developed with the ECL system (Thermo Scientific) according to manufacturer’s instructions.

### Enzyme-linked immunosorbent assay (ELISA)

ELISA plates (Nunc) were coated overnight with anti-IL-6 (Thermofisher, Cat# 16-7061-81). After blocking with 1% BSA, samples were added and incubated for 2 h at 37 °C. After washing, biotin-conjugated anti-IL-6 (Thermofisher, Cat# 36-7062-85) was added for 2 h at 37 °C followed by incubation with alkaline phosphatase-conjugated streptavidin (Southern Biotech) for 30 min at RT. The substrate p-nitrophenyl phosphate (pNPP; Sigma-Aldrich) was subsequently added and plates were read at 405 nm.

### Flow cytometry and data analysis

For surface staining, cells were resuspended in PBS 2% FCS, briefly incubated with Fc receptor blocking mAb (clone 2.4G2) and then with fluorescently labeled surface antibodies at 4 °C for 15 min. For intracellular staining, surface-stained cells were fixed with 4% formalin (10 min) and permeabilized with PBS 2% FCS 0.5% saponin. Cells were then incubated with fluorescently labeled antibodies for 30 min (room temperature). Influenza NP was stained using clone HB65 (homemade), followed by secondary staining with anti-mouse IgG-PE. Fluorescently labeled antibodies were purchased from Biolegend, ThermoFisher and BD Biosciences. After staining, cells were washed and resuspended in PBS 2% FCS for flow cytometric analysis and sorting using a FACSCalibur, a FACSCanto, a LSRFortessa and a FACSAria Fusion (all instruments from BD Biosciences). FACS data were then analyzed using FlowJo and statistical analysis was performed using Prism (Graphpad software). Throughout the article, (*) indicates a *P* value < 0.05, (**) a *P* value of < 0.01 and (***) a *P* value of <0.001.

## Supplementary information

Supplementary figureS1

Supplementary figure caption
