# Supplementary figures and images for: KappaBle fluorescent reporter mice enable low-background single-cell detection of NF-κB transcriptional activity in vivo
Source: Mucosal Immunol. 2022 May 19;15(4):656–67. doi: 10.1038/s41385-022-00525-8 (PMC9259492; doi:10.1038/s41385-022-00525-8)

Figure S1

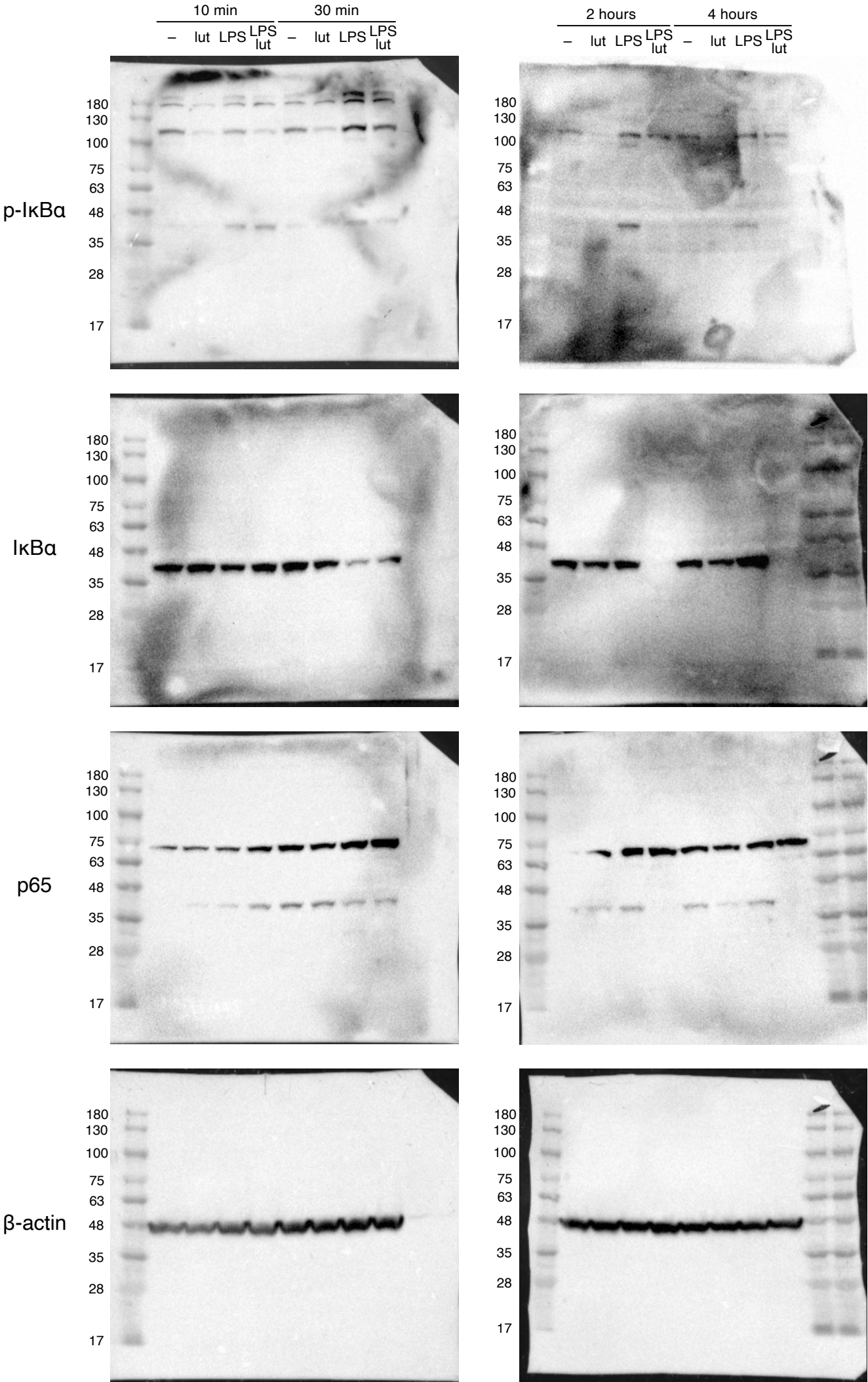

Supplement: Supplementary file 1 — Supplementary figureS1 [file 41385_2022_525_MOESM1_ESM.pdf]
