# Supplementary material for: KappaBle fluorescent reporter mice enable low-background single-cell detection of NF-κB transcriptional activity in vivo
Source: Mucosal Immunol. 2022 May 19;15(4):656–67. doi: 10.1038/s41385-022-00525-8 (PMC9259492; doi:10.1038/s41385-022-00525-8)
Supplement: Supplementary file 2 — Supplementary figure caption [file 41385_2022_525_MOESM2_ESM.docx]

**Figure S1. Uncropped images of Western blots**

Blots depict expression of IκBα (phosphoform and total), p65 and β-actin in AMs treated as indicated. Cropped images are depicted in Fig. 3J.
